# A Stroke Mimic With Postictal Todd’s Paresis and a Simplified Guide to Management of Acute Hyponatremia

**DOI:** 10.7759/cureus.44626

**Published:** 2023-09-04

**Authors:** Wenxi Tang, Dina Sulit, Mansoor Zafar, Mahmoud Abouibrahim, Abdul Paracha, Stefano Berliti, Fraser Wiggins, Periasamy Sathiskumar

**Affiliations:** 1 Internal Medicine, Conquest Hospital, East Sussex Healthcare National Health Services (NHS) Trust, Hastings, GBR; 2 Gastroenterology, Hammersmith & Charing Cross Hospitals, Imperial College London Healthcare National Health Services (NHS) Trust, London, GBR; 3 Acute Medicine, Conquest Hospital, East Sussex Healthcare National Health Services (NHS) Trust, Hastings, GBR; 4 Anaesthesiology, Conquest Hospital, East Sussex Healthcare National Health Services (NHS) Trust, Hastings, GBR; 5 Diabetes and Endocrinology, Conquest Hospital, East Sussex Healthcare National Health Services (NHS) Trust, Hastings, GBR

**Keywords:** hyponatremia, todd's paresis, postictal deficits, seizure, stroke mimic

## Abstract

Stroke mimics typically involve non-vascular disease processes, accounting for approximately half of hospital admissions for suspected stroke. These mimics may manifest as functional (conversion) disorders or indicate other neurological or medical conditions, including hypoglycemia, brain tumors, toxic poisoning, migraines, sepsis, seizures, and electrolyte imbalances, which can imitate stroke symptoms, making the diagnosis complex.

In this report, we present a unique case of a man in his sixties who developed acute postoperative hyponatremia, an electrolyte abnormality frequently encountered but rarely presented with focal neurological deficits. This condition resulted in facial asymmetry and hemiparesis; however, the remarkable outcome was that these deficits were completely resolved once the hyponatremia was corrected.

## Introduction

Diagnosing and treating stroke mimics, conditions that mimic stroke symptoms but are not caused by a blockage or bleeding in the brain, can be challenging. Common causes of stroke mimics include seizures, migraines, and psychiatric disorders [1-3]. However, certain characteristics associated with stroke mimics make it difficult to fully exclude the possibility of an actual stroke. When patients with acute neurological deficits arrive at the emergency department (ED), a rapid evaluation is crucial if intravenous tissue plasminogen activator (IV tPA) treatment is being considered. However, the short time window for administering IV tPA may not allow physicians to make a definitive diagnosis [4,5]. The evaluation typically involves a quick history, neurological examination (using the NIH Stroke Scale), and a CT scan to rule out hemorrhage. Nonetheless, this approach may miss other conditions that mimic stroke or may not reliably differentiate between conditions that mimic stroke and actual stroke.

## Case presentation

**Figure 1:**
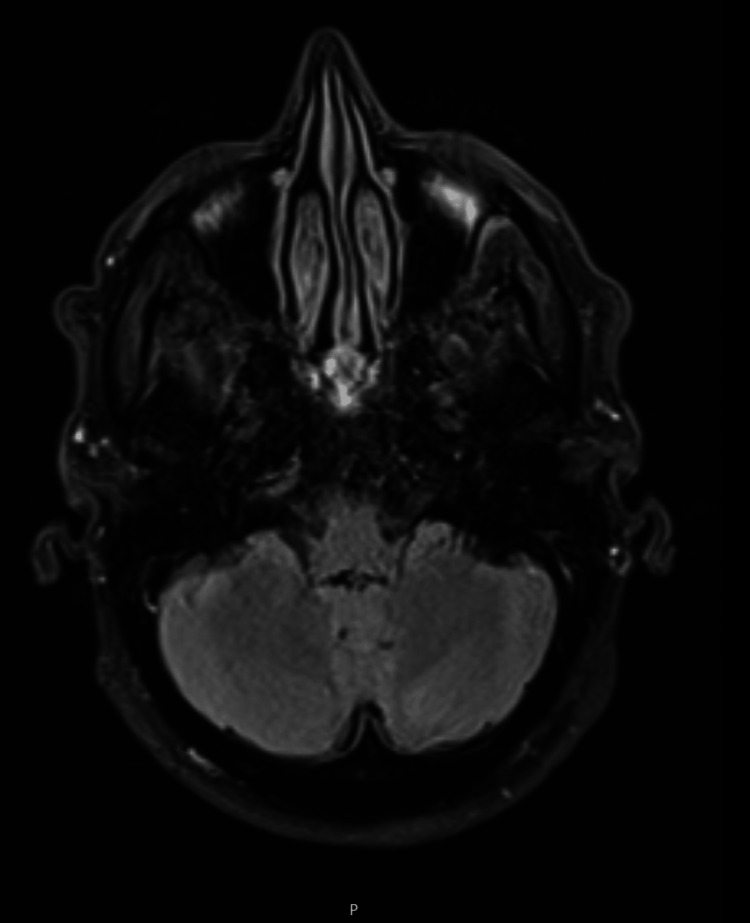
MRI brain: diffusion-weighted images with no evidence of diffusion restriction and no significant white matter abnormality.

A man in his sixties was transferred from a private hospital in the late evening hours to the ED of the district general hospital, with altered mental status, facial asymmetry, and hemiparesis, with normal healthy baselines. Medical history was significant for benign prostatic hyperplasia, and he experienced urinary urgency. Prior to the symptoms, he had a prostatic lift implant procedure during the daytime at a private hospital under general anesthetic with no complications. He was catheterized post-procedure, which was later removed in the early evening hours, and was observed to be clinically stable by the nursing team at the private hospital. A few hours later, he was subsequently found collapsed with global aphasia on the floor of the unit. He was transferred to ED with a query for acute stroke. On arrival, he had a Glasgow Coma Scale (GCS) score of 10/15, self-ventilating on room air. Venous blood gases (VBG) showed lactate of 5.8 mmol/L (reference range: 0.5-2.2), pH of 7.42, PCO_2_ of 3.9 kPa (reference range: 4.6-6.4), PO_2_ of 7.4 kPa (reference range: 11-14.4), K of 2.7 mmol/L (reference range: 3.5-5.5), glucose of 8 mmol/L (reference range: 4-7), bicarbonate of 21.2 mmol/L (reference range: 22-29), and sodium of 113 mmol/L (135-145). IV fluids and a stat dose of gentamicin were prescribed (assuming urinary tract infection with higher lactate levels on VBG) and given. The stroke team was involved, as he was assessed clinically and assigned a National Institutes of Health Stroke Scale (NIHSS) score of 21. However, thrombolysis was not attempted as he then developed a generalized tonic-clonic seizure lasting 12 seconds that self-resolved, but his GCS was noticed to have dropped to 3/15. After ensuring a secured airway, a CT scan was arranged immediately, showing no acute changes. Three hours after the first seizure, he developed another episode, which lasted around a minute. VBG showed he was profoundly acidotic with a pH of 7.07, base excess of 18 mmol/L (reference range: +2), lactate of 12 mmol/L (reference range: 0.5-2.2), and sodium of 115 mmol/L (135-145). Anti-seizure medication levetiracetam was started. His GCS was 4/15. At this point, the possibility of a stroke has not been ruled out with his initial presentation and examination. He was assumed to have a cerebrovascular event until blood results came back, which showed sodium level of 116 mmol/L (reference range: 135-145), elevated inflammatory markers with raised CRP of 22 mg/L (0-5), neutrophils of 18.49×109/L (reference range: 1.8-7.5), and WCC of 21.31×109/L (reference range: 4-11), serum cortisol of 997 nmol/L (reference range: 137-429), and serum osmolality of 248 mmol/Kg (reference range: 275-295). The impression was acute hyponatremia presenting with a stroke mimic with a complication of seizure as reflected by high lactate on VBG, low GCS, and hemiparesis. MRI brain showed no significant intracranial abnormality (Figure 1). 

He was treated with two of 150 mL boluses of 2.7% hypertonic saline aiming for a serum sodium rise of up to 5 mmol from the baseline. This resulted in an improvement of his GCS to 12/15 thirty-five minutes post-administration of hypertonic saline boluses, and he was seen freely moving both upper limbs two hours post-bolus injections. His sodium level eventually improved to 137 (Table 1 and Table 2).

**Table 1:** Lab parameters showing changes in serum sodium. Paired osmolalities and electrolytes are more suggestive of dilutional hyponatremia. *9 AM cortisol levels

Lab parameters	Units of measurement	Reference range	Labs on the day of admission	Day 2 admission	Day 4 admission	Day 6 admission
Serum sodium	mmol/L	133-146	117	118	140	137
Serum potassium	mmol/L	3.5-5.3	3.3	3.2	3.7	3.9
Urea	mmol/L	2.5-7.8	3.3	3.3	3.1	4.2
Creatinine	μmol	59-104	67	70	67	72
Corrected Ca	mmol/L	2.2-2.6	2.18	2.07	2.24	2.39
Magnesium	mmol/L	0.7-1.0	0.72	0.80	1.06	1.11
Phosphate	mmol/L	0.8-1.5	-	0.66	0.69	30
CRP	mg/L	0-5	<1	22	35	15
Haemoglobin	g/L	Male: 130-180; Female: 115-165	140	138	129	145
WCC	10^9^/L	3.6-11.0	24.54	21.31	14.25	17.77
Neutrophils	10^9^/L	1.8-7.5	22.35	18.49	11.47	14.50
Serum osmolality	mmol/Kg	275-295	248	255	276	282
Urine osmolality	mmol/kg	300-900	110	113	226	367
Urine sodium	mmol/L	<30	<20	<20	<20	-
Urine potassium	mmol/L	0-10	14	6	4	-
Serum cortisol	nmol/L	137-429	-	997^*^	-	-
Serum thyroid stimulating hormone	mIU/L	0.27-4.20	0.90	-	-	-

**Table 2:** A detailed periodic serum sodium correction throughout the hospital stay.

Lab parameters	Units	Ref	day 1 21:00	Day 1 22:30	Day 2 10:30	Day 2 11:00	Day 2 15:30	Day 2 19:30	Day 2 22:30	Day 3 07:00	Day 3 15:30	Day 3 22:30	Day 4 01:00	Day 4 06:00	Day 5 02:30	Day 5 07:00	Day 6 10:45	Day 7 08:45	Day 7 17:00	Day 8 08:00
Serum sodium	mmol/L	133-146	117	115	118	118	123	128	132	134	138	138	138	140	138	141	137	137	137	137

During the ward round the next day, his GCS was 15/15, and serum sodium was 137 mmol/L with no impairment in speech or mobility. At this moment, a discussion with the patient revealed that he was concerned about transient hematuria via urinary catheter post-procedure and was encouraged by the urologist to have moderate oral fluids so as to wash away with a hopeful resolution of the symptoms.

The patient, however, following this discussion overdid his intake of water and had about 5 L of water in the next four to five hours followed by headache and some dizziness that he attributed at that time to a possible post-general anesthetic effect. He aimed to drink more water so as to feel better and chose to ignore mentioning to the nursing team. An impression of dilutional hyponatremia was reflected in paired osmolality and electrolytes and seizure due to hyponatremia that was reflected with raised lactate and stroke mimics presenting as postictal Todd’s paresis. He eventually made clinical improvements and was deemed medically fit for discharge with outpatient clinic follow-up by the endocrinology and neurology teams, while being on Levetiracetam.

## Discussion

Hyponatremia is the most prevalent electrolyte disorder, impacting around 5% of adults and 35% of hospitalized patients. It is characterized by a serum sodium level below 135 mEq/L and is primarily caused by water retention [6]. Hyponatremia can present with a wide range of symptoms, e.g., difficulty concentrating, nausea, forgetfulness, apathy, loss of balance, and in severe cases, it can lead to seizures and coma [7,8]. As demonstrated in this case report and other published literature, stroke mimic symptoms (e.g., focal unilateral neurologic deficit and aphasia) can also be secondary to acute hyponatremia [8,9].

Both hyponatremia and stroke are common in the elderly and as high as 30% of patients presenting in the ED with symptoms suggestive of acute stroke are stroke mimics [9]. Brunser et al. highlighted that aphasia is the most common symptom in hyponatremia stroke mimics (37.6%) [10]. The acute osmotic fluid shift intracerebrally and subsequent brain edema are the underlying mechanisms of neurologic deficit in hyponatremia [11].

Diagnosing a stroke mimic secondary to underlying acute hyponatremia is a challenge as the risk of missing significant disabling real stroke could be catastrophic, but on the other hand, giving tPA might be unnecessary. Rapid assessment is required to assess any patient with a potential ischemic cerebral event, especially within the thrombolysis window. Factors favoring stroke mimics include seizures at onset, lack of lateralization, signs or symptoms not attributed to specific vascular territory, and other co-existing signs and symptoms not explained by stroke, i.e., chest symptoms and a lack of cardiovascular risk factors like hypertension, diabetes, ischemic heart disease, TIAs, previous stroke, or AF. Urgent imaging, particularly MRI, is necessary for diagnosis, and improved access to emergency MRI services could potentially decrease the need for hospital admissions and lead to reduced length of stay (LOS) [12].

Reassuringly, studies showed a small percentage of complications in those who received tPA for stroke mimics, and therefore, it is preferable to give thrombolysis rather than to delay treatment in cases with a high probability of acute stroke [13].

Acute hyponatremia is not only a potential cause of stroke mimicking but also it is a common association with stroke (up to 54%) and has been associated with adverse outcomes and increased short-term and long-term mortality [14,15]. Prompt diagnostic assessment of hyponatremia is crucial when encountering individuals displaying acute hyponatremia. This assessment encompasses obtaining a detailed medical history, conducting a thorough physical examination, and initiating laboratory analyses including serum osmolality, urine osmolality, urine sodium concentration, and serum levels of other electrolytes [16]. Furthermore, a comprehensive hormonal assessment, including measurement of cortisol and thyroid-stimulating hormone, and paired serum, urine osmolality and electrolytes should be included. 

Treatment for hyponatremia stroke mimic is mainly achieved by correcting the low sodium through achieving euvolemia and controlling the causative agent, such as stopping the culprit diuretic or proton pump inhibitor and investigating and treating the underlying pathology leading to conditions, e.g., SIADH and renal sodium loss. In cases of acute severe hyponatremia, characterized by a rapid decline in serum sodium levels over a period of less than 48 hours and accompanied by neurological manifestations, the recommended approach involves correcting sodium concentrations by administering hypertonic saline (3%) boluses. It is imperative to closely monitor sodium levels during this process, with the objective of not exceeding an increase of more than 10 mmol/L within 24 hours to avoid cerebral dehydration and osmotic demyelination syndrome [14,17]. If there is an improvement in symptoms following a rise of 5 mol/L in serum sodium levels from the baseline, it is advisable to discontinue the 3% bolus hypertonic saline and initiate treatment tailored to the specific cause, aiming to sustain the achieved serum sodium concentration [18].

**Figure 2:**
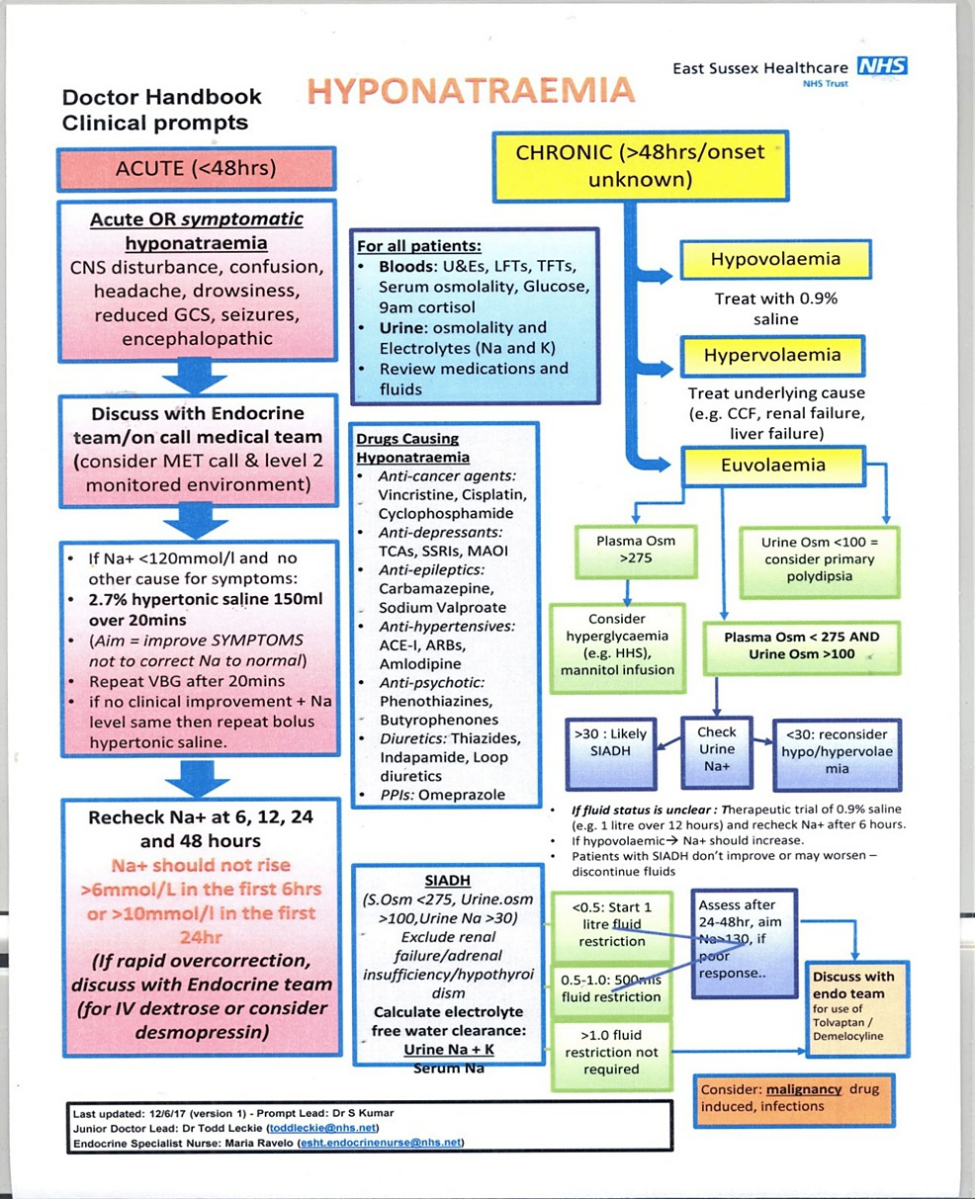
A simplified guide to diagnosing and managing patients with acute hyponatremia. Permission to reproduce the image obtained from Dr. Sathis Kumar, Clinical Lead of the Department of Endocrinology and Diabetes at East Sussex Healthcare NHS Trust, United Kingdom [19].

A very helpful guide for diagnosing and correcting hyponatremia is outlined in a guide from East Sussex Healthcare NHS Trust (Figure 2) [19].

Careful monitoring of serum sodium levels within the initial 24 hours of treatment is recommended, followed by daily monitoring thereafter to ensure a gradual and controlled increase in serum sodium concentrations. Adjustment of treatment protocols based on individual patient response is a crucial measure in mitigating the risk of inadvertent overcorrection [18,20]. As shown in this case and other published literature, the neurological manifestations due to hyponatremia are reversible within days with favorable outcomes [8,9,13].

Finally, there are multiple published outcomes for Todd’s paresis including following vasovagal syncope and from the electrolyte disturbances including hypomagnesemia toxic poisoning from methyl iodide, and also a patient with paresis and hyponatremia with MRI evidence of cortical changes that reversed post thrombolytic management followed by later onset of focal seizures that seem to respond to correction of hyponatremia but, to the best of our knowledge we present here a unique case of postictal Todd’s paresis associated with dilutional hyponatremia where seizures due to hyponatremia led to postictal paresis with no CT and/or MRI evidence of any stroke changes [21-24].

Considering that reported stroke mimics account for approximately half of the hospital admissions for suspected stroke, it will be interesting to see more case reports in the future toward the exact prevalence [25]. Additionally, we hope the guidelines for hyponatremia management will be helpful for the prompt and timely management of patients presenting with severe hyponatremia in acute settings. 

## Conclusions

Stroke mimics including postictal Todd’s paresis can pose challenges in emergencies and may initially be treated as an actual stroke. This case report highlights the importance of remaining vigilant about conditions that can imitate acute stroke, such as hyponatremia. It emphasizes the need to gather a detailed patient history and commence active investigations from the beginning of the patient presentation. Conducting a timely and thorough evaluation is crucial since many of these stroke mimics are reversible, and with appropriate correction, positive outcomes can be achieved.
